# Sensitivity of Bovine Tuberculosis Surveillance in Wildlife in France: A Scenario Tree Approach

**DOI:** 10.1371/journal.pone.0141884

**Published:** 2015-10-30

**Authors:** Julie Rivière, Yann Le Strat, Barbara Dufour, Pascal Hendrikx

**Affiliations:** 1 Research unit EpiMAI USC Anses (Epidemiology of Animal Infectious Disease), Alfort National Veterinary School, Maisons-Alfort, France; 2 Department of Infectious Diseases, French Institute for Public Health Surveillance, Saint-Maurice, France; 3 Unit UCAS, French Agency for Food, Environmental and Occupational Health and Safety (Anses), Maisons-Alfort, France; INIAV, I.P.- National Institute of Agriculture and Veterinary Research, PORTUGAL

## Abstract

Bovine tuberculosis (bTB) is a common disease in cattle and wildlife, with an impact on animal and human health, and economic implications. Infected wild animals have been detected in some European countries, and bTB reservoirs in wildlife have been identified, potentially hindering the eradication of bTB from cattle populations. However, the surveillance of bTB in wildlife involves several practical difficulties and is not currently covered by EU legislation. We report here the first assessment of the sensitivity of the bTB surveillance system for free-ranging wildlife launched in France in 2011 (the Sylvatub system), based on scenario tree modelling. Three surveillance system components were identified: (i) passive scanning surveillance for hunted wild boar, red deer and roe deer, based on carcass examination, (ii) passive surveillance on animals found dead, moribund or with abnormal behaviour, for wild boar, red deer, roe deer and badger and (iii) active surveillance for wild boar and badger. The application of these three surveillance system components depends on the geographic risk of bTB infection in wildlife, which in turn depends on the prevalence of bTB in cattle. We estimated the effectiveness of the three components of the Sylvatub surveillance system quantitatively, for each species separately. Active surveillance and passive scanning surveillance by carcass examination were the approaches most likely to detect at least one infected animal in a population with a given design prevalence, regardless of the local risk level and species considered. The awareness of hunters, which depends on their training and the geographic risk, was found to affect surveillance sensitivity. The results obtained are relevant for hunters and veterinary authorities wishing to determine the actual efficacy of wildlife bTB surveillance as a function of geographic area and species, and could provide support for decision-making processes concerning the enhancement of surveillance strategies.

## Introduction

Bovine tuberculosis (bTB) is a chronic disease caused by *Mycobacterium bovis* or, less frequently, by *M*. *caprae*. It affects livestock species, especially cattle, but also companion and wild animals, and it may cause zoonotic disease in humans [1]. In developed countries, bTB results in major economic losses in the livestock sector, with costs to the cattle industry and government due to surveillance expenses (testing costs), movement restrictions and compensation for slaughtered cattle.

Infected wild animals have been detected in some European countries and wildlife reservoirs have been identified [2, 3]. Wild species, including maintenance hosts in particular, represent a major obstacle to the eradication of bTB in cattle, because they constitute a potentially continuous source of re-infection [3, 4, 5]. A good understanding of the ecology and behaviour of relevant species and their role in bTB epidemiology is therefore essential, to evaluate the potential for transmission to the cattle population and for the implementation of effective disease control measures [3, 6, 7]. There is currently no EU legislation relating to bTB surveillance and control programs for wildlife, but some countries have implemented a national surveillance programme [8].

The European Commission has considered France to be bTB-free since 2000. However, several infected herds are still detected each year in some areas. The first cases of bTB in wildlife were detected in red deer (*Cervus elaphus*) and Eurasian wild boar (*Sus scrofa*) in 2001. Additional cases have been detected regularly ever since, in wild boar, red deer and badgers (*Meles meles*) in the vicinity of cattle outbreaks, but the role of susceptible wild animal species in bTB epidemiology remains unclear [9]. A national surveillance programme for bTB in wildlife, the Sylvatub programme, was launched in France in 2011. The main aims of this surveillance system are the early detection of cases and the monitoring of infection levels in affected areas. It consists of three independent surveillance system components (SSCs): (1) passive scanning surveillance on hunted wild boar, red deer and roe deer; (2) passive surveillance on animals found dead, moribund or with abnormal behaviour, for wild boar, red deer, roe deer and badger; (3) active surveillance on hunted wild boar and trapped badger. Passive scanning surveillance is defined as the reporting of suspect cases by stakeholders, whereas active surveillance is defined as the collection of samples according to a predetermined sampling framework.

However, bTB surveillance in wildlife species is subject to several practical difficulties (geographic dispersion of populations, unknown population density, difficulties observing and collecting animals, non-random selection of the most accessible animals, leading to potential sampling biases) and is generally driven by ecological, economic and/or social (political, cultural) considerations, as most of the stakeholders involved in the French surveillance system are volunteers. Furthermore, surveillance in wildlife is based principally on post-mortem examinations, but several studies have shown that the detection of tuberculosis-like lesions by field stakeholders has a low sensitivity, leading to an underestimation of the prevalence of bTB in wildlife species [10, 11, 12]. There is therefore a need to assess the efficacy of the wildlife surveillance system implemented in France.

We present here the first assessment of the sensitivity of the bTB surveillance system in free-ranging wildlife in France (Sylvatub), using scenario tree modelling. This method makes it possible to quantify the sensitivity of a component of a surveillance system based on non-probabilistic sampling [13], defined as the probability of detecting at least one infected animal in a population with a given design prevalence. The bTB surveillance components were evaluated for each species separately.

## Materials and Methods

### 2.1. Ethics statement

This study did not involve the deliberate killing of animals for the sole purpose of the study, as all samples were collected from animals trapped or hunted legally during the hunting season with appropriate permits, shot legally because of severe debilitation or found dead. All the samples included in this study were obtained from animals analyzed within an official context relating to bTB surveillance in free-ranging wildlife. All sampling procedures complied with national and European regulations and no specific ethics approval was therefore required.

### 2.2. The French bTB surveillance system for free-ranging wildlife

**Fig 1:**
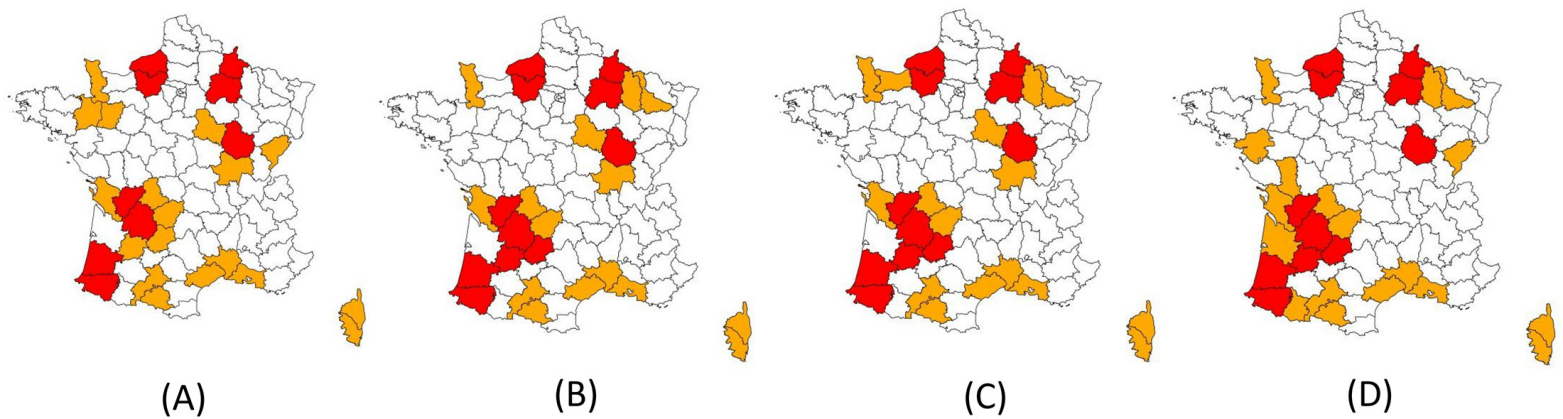
Geographical risk levels for the surveillance of bTB in wildlife in France from 2013 to 2015. Low-risk level: white, Medium-risk level: orange; High-risk level: red. From left to right: (A) July 2013, (B) January 2014, (C) July 2014 and (D) January 2015.

The French surveillance system for bTB in wildlife consists of three independent SSCs described below. These SSCs are applied as a function of geographic risk, which is assessed on the basis of outbreaks in cattle or wildlife. Local risk is regularly re-evaluated, depending on changes in the epidemiological situation in cattle and wildlife (Fig 1A–1D). Three levels of risk have been defined [14]:

- High-risk areas, with several outbreaks in cattle or wildlife (threshold of 10 bovine outbreaks in two years in the same area): this level of risk is applied for several years, to monitor infection levels in wildlife and to assess the efficacy of control measures;
- Medium-risk areas, with some outbreaks in cattle, resulting in a higher increased incidence of the disease, and/or areas in close geographic proximity to high-risk areas (neighbouring “*départements*”, the adjacent administrative areas): this level of risk is applied for at least one year and for as long as necessary to obtain a good understanding of the epidemiological situation. A low- or high-risk level is subsequently applied, depending on the surveillance results;
- Low-risk areas, with few TB cattle outbreaks, if any, and no increase in incidence.

#### 2.2.1. Passive scanning surveillance on hunted wild animals by carcass examination (EC-SSC)

This surveillance component applies to hunted wild boar, red deer and roe deer and is based on post-mortem examination and the voluntary submission, by hunters, of carcasses with macroscopic tuberculosis-like lesions (TBLs). In all geographic areas (*i*.*e*. regardless the local risk), hunters are asked to submit any game animals with macroscopic tuberculosis-like lesions (TBLs) to laboratories for testing, free of charge. The hunter’s awareness of macroscopic TBLs is crucial for their detection and, therefore, for the sensitivity of the EC-SSC [15]. Since the initial implementation of Sylvatub in 2011, hunters have become more aware of bTB, due to the growing number of articles in hunting magazines, and the implementation of training and information campaigns in high-risk regions. Furthermore, some hunters take specific training courses, to learn how to detect abnormal carcasses (with lesions), but this training is not mandatory.

#### 2.2.2. Surveillance on animals found dead, moribund or with abnormal behaviour (SAGIR-SSC)

Passive surveillance on dead and dying animals, through the SAGIR network, has been implemented in France since 1986. This surveillance component relies on field stakeholders (hunters, local hunting federations and technicians from the National Hunting and Wildlife Office) providing an inventory of dead or moribund animals (red and roe deer, wild boar, badger) found in forests or at the roadside and bringing these animals to a laboratory for investigations at their own expense, through their local federations (the analysis depends on the results of the necropsy) [16]. Within the Sylvatub national surveillance programme, laboratory partners are asked to report bTB results and the SAGIR network receives assistance, free of charge, in areas of medium or high risk (assistance with the collection of animals by field stakeholders, particularly for large animals, and systematic bTB analysis, even in the absence of TBL detection on necropsy).

#### 2.2.3. Active surveillance (PSURV-SSC)

Systematic bTB analysis (see 2.2.4) is conducted on a planned sample of 15 badgers trapped within a radius of 1 km around outbreaks of bTB in cattle in medium-risk areas, and on samples of a hundred badgers and a hundred wild boars in larger areas within high-risk zones, according to the geographic distributions of these species. Animals are collected even if no macroscopic TBLs are detected by field stakeholders. In these high-risk areas, the aim is to detect bTB infection, assuming a prevalence of 3%, with a 95% confidence level.

#### 2.2.4. Tissue collection and laboratory investigations

Field stakeholders submit animals, organs or tissues (from a standardized list of samples) to a laboratory immediately after collection, with a data sheet containing information about the species, estimated age (juvenile or adult), sex, date and location of collection, and body condition (degradation, presence of TBL on the carcass, etc.). Some field stakeholders have been trained in organ collection through practical exercises on carcasses. For wild boar and deer, the organs of the pulmonary and digestive systems are collected, for examination of the tracheobronchial, mediastinal and mesenteric lymph nodes, together with the head, for examination of the retropharyngeal and mandibular lymph nodes, and with any organs presenting lesions. For badgers, which are relatively small, the entire animal is often collected.

A necropsy is carried out, together with a detailed analysis of the organs collected, by qualified laboratory staff, for the detection of TBLs (caseo-granulomas, mineralised nodules or purulent abscesses [17, 18, 19, 20]). The diagnostic process differs between SSCs:

- For EC-SSC at all risk levels and for SAGIR-SSC in low-risk areas, samples are analysed by culture and PCR only if TBLs are detected by the laboratory staff;
- For SAGIR-SSC and PSURV-SSC in medium- and high-risk areas, the samples from collected animals are systematically cultured, and PCR is also conducted on the TBLs, if present (pooled samples).

If a non-negative result is obtained at a local laboratory, biological material is sent to the national reference laboratory for confirmation. The bacterial culture was performed following the protocol established by the French NRL for isolation of *M*. *bovis* (culture on solid media after decontamination) [21]. The PCR relies on a commercial kit (the targeted sequence was IS6110, present in all species of the M. tuberculosis complex) [21].

### 2.3. Scenario trees and model description

bTB surveillance in free-ranging wildlife cannot be evaluated by traditional methods involving data collection by random sampling methods [22, 23]. Scenario tree models have been developed for the quantitative evaluation of complex veterinary surveillance systems [13, 24]. The sensitivity of detection of a surveillance system is defined as the probability of detecting at least one animal positive for bTB for a given prevalence in the population. A tree represents all the events influencing the detection of the infection as nodes dividing the population into groups of animals with similar probabilities of being infected and detected. Each of these category nodes may have one or more possible outcomes, with a specific probability of occurrence estimated from historical data, published findings or expert opinion. The final outcome of each pathway of the tree is obtained by multiplying all the probabilities along the limb.

**Fig 2:**
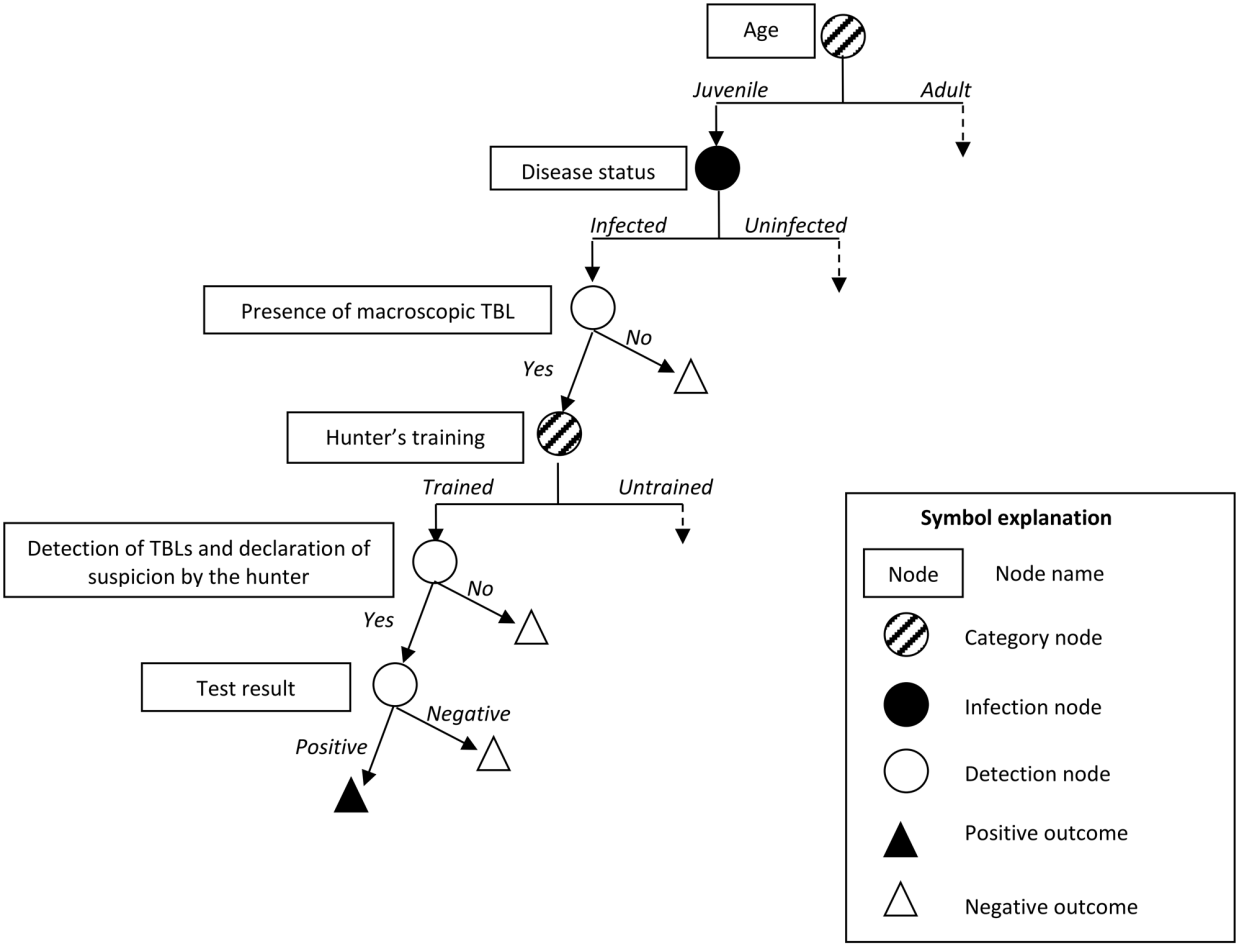
Scenario tree illustrating the scanning surveillance system component based on carcass examination for hunted wild boar, red deer and roe deer (EC-SSC, applied in areas of all risk levels).

**Fig 3:**
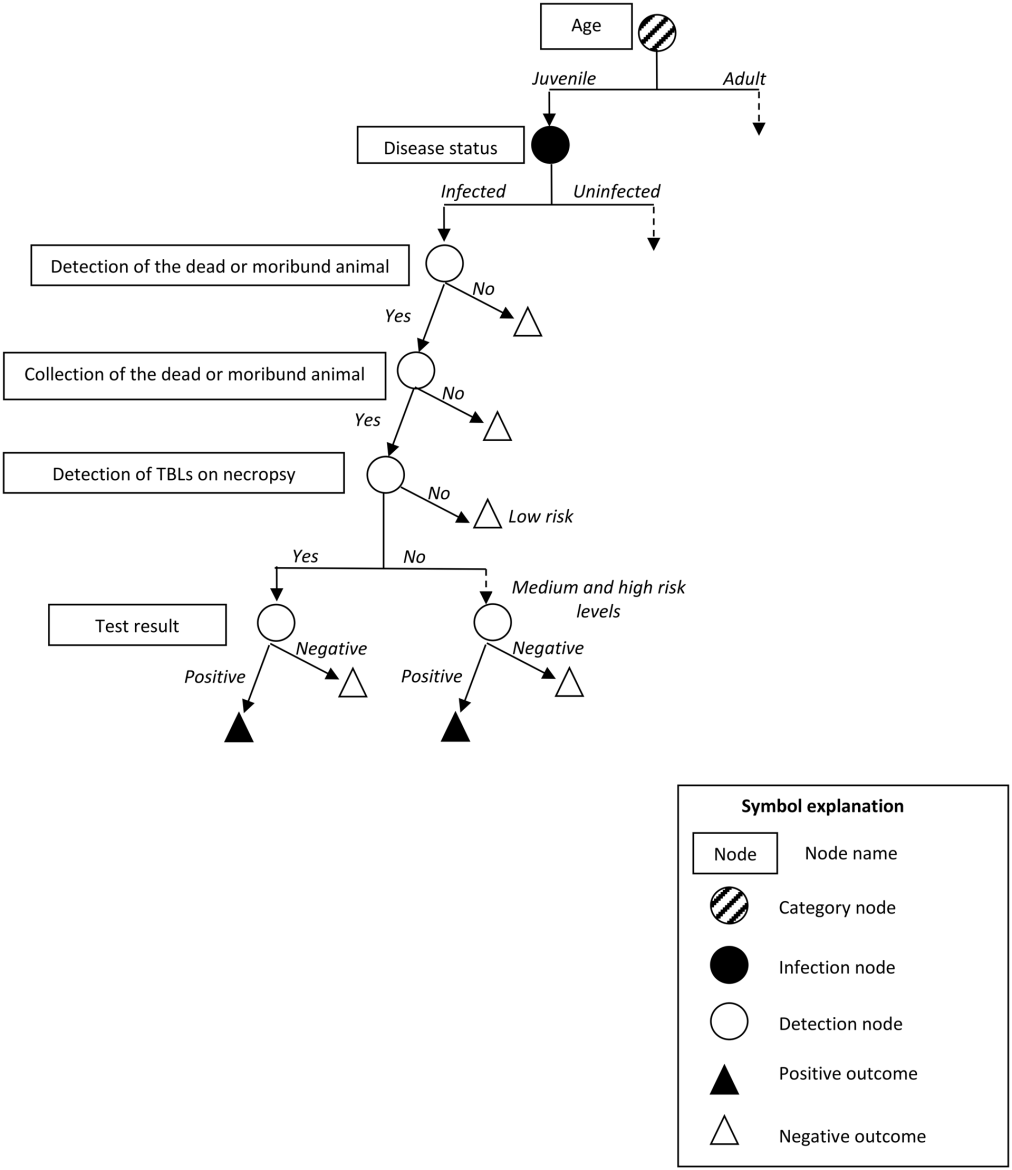
Scenario tree illustrating the surveillance system component for animals found dead, moribund or with abnormal behaviour (SAGIR-SSC, applied to each species and all risk levels).

**Fig 4:**
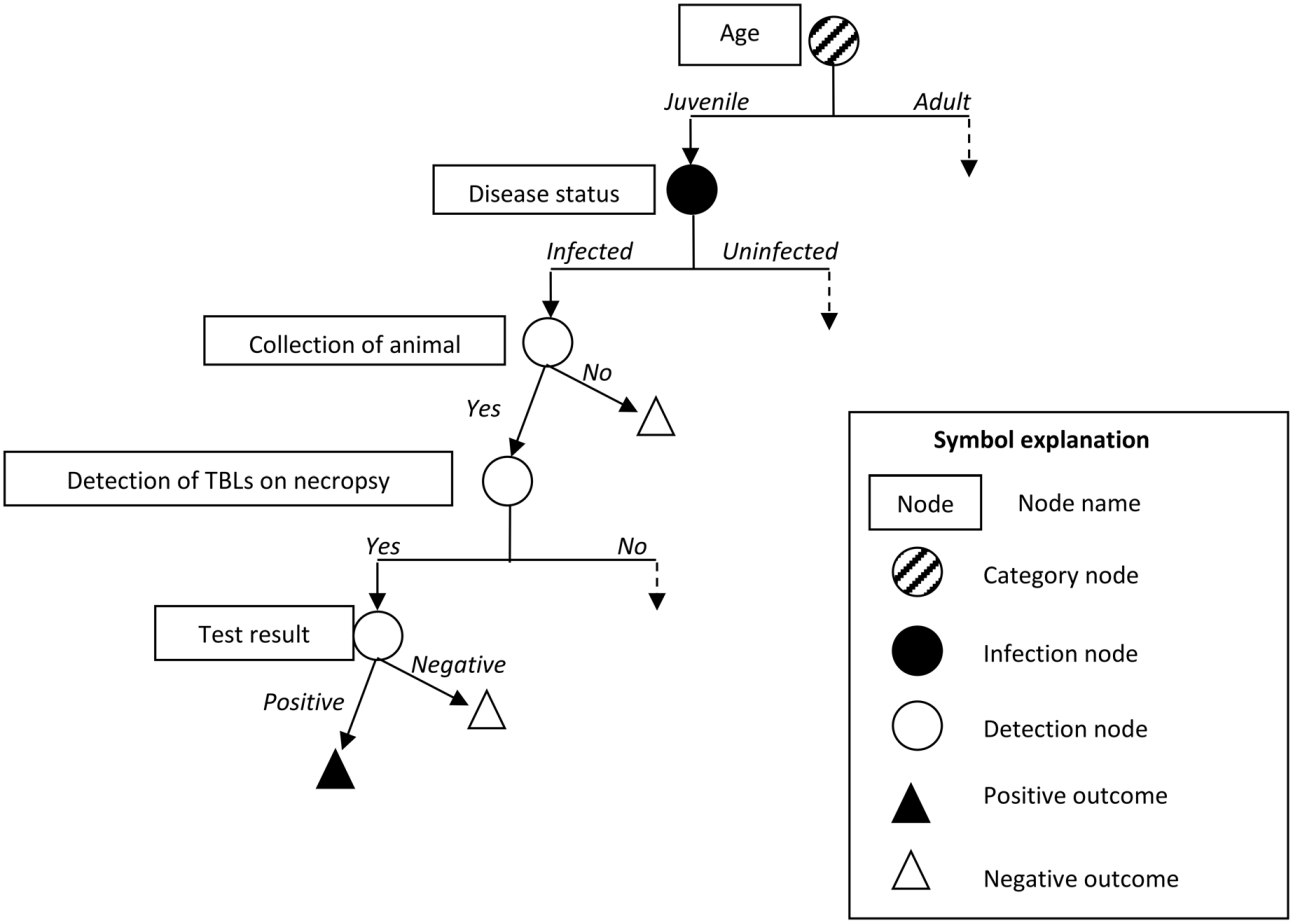
Scenario tree illustrating the active surveillance system component for badger and wild boar in medium- and high-risk areas (PSURV-SSC).

This methodology was applied to each of the three SSCs of the French surveillance system over a one-year period, as bTB is a chronic disease, and the SSCs were evaluated for each species separately. The structure and content of the scenario trees (nodes and input parameters) are described below and illustrated in Figs 2–4. The parameters of the stochastic model were estimated from data for bTB surveillance in France for the 2013–2014 hunting season, but also from published results and expert opinion, because the Sylvatub data are not entirely accurate and complete, as this surveillance system was only recently implemented and wildlife monitoring is difficult. We used a Delphi approach to collect expert opinions, so as to obtain consensus minimum, maximum and most likely values for the probability of a wild animal having TBLs, the probability of such TBLs being detected by a hunter and the probability of a dead or moribund wild animal being detected and collected by field stakeholders. An electronic questionnaire was sent to 16 French experts in the field of veterinary and wildlife epidemiology, hunters’ associations and the SAGIR network, for the individual assignment of values. The results were then presented to the experts, to allow them to change their minds if they felt they had overestimated or underestimated the values when they first completed the questionnaire. A description of the experts contacted and the questionnaire sent to them are provided in the supplementary material (S1 File). Input parameters were described by distributions, to account for the uncertainty of the estimates.

For each species and risk level, the mean and its 95% confidence interval were estimated from 10,000 simulated values, by a Monte Carlo method. The scenario trees were implemented in Excel and the Monte Carlo method, with a fixed random seed of one, was run stochastically with @RISK (Decision Tool, version 6).

#### 2.3.1. Model coverage

Scenario trees were modelled by SSC, geographic risk and species, with the age class as a category node. This made it possible to split the wildlife population into several homogeneous groups, according to the probabilities of infection and detection. We identified values of the Pert distributions through a literature review; subadults were grouped with the adult class for wild boars and with the juvenile class for deer, in accordance with expert opinion (Table 1).

**Table 1:** Proportion of juveniles and adults in the wildlife population according to species.

Species	Age class	Proportion in the wild population
Wild boar	Juvenile	Pert (0.50; 0.55; 0.60) [25, 26]
	Adult	1 –P(juveniles)
Deer (red and roe deer)	Juvenile	Pert (0.20; 0.24; 0.35) [27, 28]
	Adult	1 –P(juveniles)
Badger	Juvenile	Pert (0.25; 0.40; 0.50) [29, 30]
	Adult	1 –P(juveniles)

#### 2.3.2. Design prevalence and probability of infection

The sensitivity of a SSC applied to a population depends on the prevalence of the disease in this population, referred to as the “design prevalence” or “threshold prevalence” (denoted P*). The design prevalence is usually set according to international standards or trading requirements and is unrelated to the real prevalence in the study population, which is usually assumed to be zero [13]. It is the lower limit of the theoretical prevalence of infection in the population detectable by the surveillance system with a specified probability [13] and should be assigned to the infection node. However, there is currently no standard design prevalence for bTB surveillance in wildlife. The surveillance system should be able to detect a prevalence of 3% with a confidence level of 95% in high-risk areas. The adjusted risk of infection is usually based on the relative risk and the proportion of the population in each risk group [13]: as they are not accurately known in the wild populations, we used the prevalence in cattle in the three risk areas to estimate the relative risk of infection for wild species for each risk level (the risk level for the Sylvatub System was determined from epidemiological data for bTB in cattle, as there is a good correlation between outbreaks in wild animals and in cattle [9]). Based on the prevalence data for cattle, the relative risks of infection in wildlife in medium- and high-risk areas were 13 and 38, respectively, with the low-risk area used as the reference. From the prevalence of 3% in high-risk areas and these relative risks, we deduced the expected probabilities of infection in low- and medium-risk areas. Then for each risk level, a multivariate Poisson regression was performed to predict the probabilities of infection depending on species and age. Finally, for each specie and age, we obtained adjusted probabilities of infection (Table 2) by dividing the regression-based probabilities of infection by the expected probabilities of infection in each level.

**Table 2:** Probability of infection in scenario trees according to species, age class and geographic risk.

Species	Age class	Low risk level (%)	Medium risk level (%)	High risk level (%)
Wild boar	Juvenile	0.06	0.79	2.26
	Adult	0.08	1.14	3.26
Red deer	Juvenile	0.01	0.16	0.46
	Adult	0.02	0.23	0.66
Roe deer	Juvenile	0.06	0.84	2.40
	Adult	0.09	1.21	3.47
Badger	Juvenile	0.07	0.93	2.67
	Adult	0.10	1.35	3.85

#### 2.3.3. Diagnostic process

The sensitivity of culture and PCR on wild populations are not accurately known actually. They have been estimated in France, on cattle populations, by latent class analysis, on samples with bTB compatible lesions and from herds with a bTB suspicion [21]. However, samples from wild animals are of lower quality than those from cattle, due to degradation and the bacteriological contamination of carcasses or samples. This would probably decrease culture sensitivity by about 35% (expert opinion). Furthermore, pooled samples were analysed for wildlife, whereas cattle samples were analysed separately. This may have decreased the sensitivity of the PCR test by about 15% (expert opinion). We therefore applied a modulating factor when estimating the sensitivity of tests on wildlife: the sensitivity of the culture and PCR methods for wildlife were estimated at 50.8% [47.4%; 53.8%] and 74.45% [70.1%; 78.5%], respectively, and modeled by a Pert distribution.

If TBLs were detected, culture and PCR tests were carried out in parallel at the local laboratory. Assuming the conditional independence of tests, sensitivity at the local laboratory was calculated as follows: *Se*_*locallab*_ = 1−(1−*Se*_*culture*_)×(1−*Se*_*PCR*_). If positive results for culture and/or PCR were obtained at the local laboratory, a discriminating PCR was carried out, in series, at the national laboratory for confirmation. The sensitivity of this test was estimated at 90% [85%; 95%] (expert opinion). The sensitivity of the whole diagnostic process was calculated as follows: *Se*_*whole*_ = *Se*_*locallab*_×*Se*_*PCRnational lab*_.

#### 2.3.4. Specific parameters for passive scanning surveillance on hunted wild animals (EC-SSC; Fig 2)

The main detection nodes influencing the probability of bTB detection by the EC-SSC are the presence of macroscopic TBLs, and the detection of these TBLs by a hunter, who subsequently reported his suspicions, triggering the diagnostic process (Fig 2). The probability of TBL being present on an infected animal with *M*. *bovis* depends on the species and age of the animal. It was estimated by an expert panel and was modelled with a Pert distribution (Table 3). The likelihood of detecting TBLs depends on the awareness of hunters and the species hunted, as the patterns of lesions differ between wildlife species [20]. Assumptions about disease awareness were based on the training and experience of the hunters (as a function of the risk in their local administrative area (*département*)). The probabilities of TBLs being detected by a hunter on post-mortem inspection and of laboratory confirmation being requested were estimated together, by the expert panel, for each species and hunter’s level of awareness (Table 4).

**Table 3:** Probability of a wild infected animal displaying macroscopic TBLs, as a function of the species concerned and age class.

Species	Age class	Values
Wild boar	Juvenile	Pert (0.40; 0.50; 0.90)
	Adult	Pert (0.50; 0.50; 0.90)
Red deer	Juvenile	Pert (0.60; 0.90; 1)
	Adult	Pert (0.60; 0.85; 1)
Roe deer	Juvenile	Pert (0.50; 0.80; 0.90)
	Adult	Pert (0.50; 0.80; 0.95)

**Table 4:** Probability of a hunter detecting macroscopic TBLs, by species and awareness of the hunter (training and risk level).

Hunter’s training status	Species	Risk level		
		Low risk level	Medium risk level	High risk level
Trained	Wild boar	Pert (0.05; 0.30; 0.50)	Pert (0.10; 0.40; 0.90)	Pert (0.40; 0.80; 0.95)
	Red deer	Pert (0.05; 0.70; 0.85)	Pert (0.40; 0.70; 0.90)	Pert (0.50; 0.90; 1)
	Roe deer	Pert (0.20; 0.60; 0.80)	Pert (0.40; 0.50; 0.90)	Pert (0.60; 0.90; 1)
Untrained	Wild boar	Pert (0; 0.10; 0.30)	Pert (0; 0.25; 0.50)	Uniform (0; 1)
	Red deer	Pert (0; 0.25; 0.50)	Uniform (0; 0.75)	Uniform (0; 0.90)
	Roe deer	Pert (0; 0.25; 0.50)	Uniform (0; 0.75)	Pert (0.30; 0.50; 0.90)

#### 2.3.5. Specific parameters for passive surveillance on animals found dead, moribund or with abnormal behaviour (SAGIR-SSC; Fig 3)

The detection of an infected wild animal by the SAGIR-SSC depends on the probability of a dead or moribund animal being collected by a stakeholder in the field (Table 5, estimated from expert opinion) (Fig 3). The ability to detect and collect a dead or moribund animal depends on its size and, consequently, on its species and age. The collection of such animals is also dependent on the level of risk, through the field partner’s level of awareness and economic considerations (analyses are paid for by the hunter’s association in low-risk areas, but are reimbursed in medium- and high-risk areas).

**Table 5:** Probabilities of a dead or moribund wild animal being detected and collected by a field partner.

Species	Age	Detection	Collection		
			Low risk level	Medium risk level	High risk level
**Wild boar**	Juvenile	Pert (0.05; 0.15; 0.40)	Pert (0; 0.05; 0.30)	Pert (0.01; 0.20; 0.50)	Uniform (0.10; 0.90)
	Adult	Pert (0.05; 0.15; 0.40)	Pert (0; 0.03; 0.20)	Pert (0,03; 0,10; 0,40)	Uniform (0,10; 0,80)
**Red deer**	Juvenile	Pert (0.05; 0.20; 0.60)	Pert (0; 0.05; 0.30)	Pert (0.01; 0.30; 0.75)	Pert (0.35; 0.50; 1)
	Adult	Pert (0.05; 0.20; 0.60)	Pert (0; 0.05; 0.30)	Uniform (0.03; 0.80)	Pert (0.20; 0.50; 1)
**Roe deer**	Juvenile	Pert (0.05; 0.15; 0.40)	Pert (0; 0.05; 0.10)	Pert (0.01; 0.20; 0.30)	Uniform (0; 1)
	Adult	Pert (0.05; 0.15; 0.40)	Pert (0; 0.05; 0.10)	Uniform (0; 1)	Uniform (0; 1)
**Badger**	Juvenile	Pert (0.01; 0.20; 0.40)	Pert (0; 0.02; 0.20)	Pert (0; 0.05; 0.50)	Pert (0.10; 0.50; 0.70)
	Adult	Pert (0.01; 0.20; 0.40)	Pert (0; 0.05; 0.20)	Pert (0.10; 0.50; 0.60)	Pert (0.10; 0.50; 0.70)

Once collected, the carcasses are taken to a local laboratory for necropsy. In low-risk areas, further analyses are conducted only if TBLs are detected by qualified staff. By contrast, in medium- and high-risk areas, in which surveillance measures are more stringent, bacteriological culture is systematically carried out for all samples, even if no TBL is detected on necropsy. The probability of a wild animal having TBLs detected at the laboratory depends on a number of factors, including the infectious status, species and age of the animal. We performed multivariate logistic regression analyses on current surveillance system data for France, from which we calculated the probability of a wild animal having TBLs detected at the laboratory (dependent variable), as a function of infectious status, species and age class (explanatory variables). Then we computed the predictions and their standard deviations from the model, giving the parameters of the Gaussian distributions presented in Table 6 to modeled the probability of TBLs being detected on an infected animal at the laboratory by qualified staff, by species and age class.

**Table 6:** Probability of TBLs being detected on an infected animal at the laboratory by qualified staff, by species and age class.

Species	Age class	Values
Wild boar	Juvenile	Normal (0.58; 0.029)
	Adult	Normal (0.61; 0.016)
Red deer	Juvenile	Normal (0.44; 0.040)
	Adult	Normal (0.47; 0.034)
Roe deer	Juvenile	Normal (0.88; 0.017)
	Adult	Normal (0.89; 0.011)
Badger	Juvenile	Normal (0.40; 0.032)
	Adult	Normal (0.43; 0.011)

#### 2.3.6. Specific parameters for active surveillance (PSURV-SSC; Fig 4)

Active surveillance is dependent on fewer factors, as a predetermined number of animals should be collected and analysed, even if no macroscopic or microscopic TBLs are detected (Fig 4). The main factor likely to influence the sensitivity of the PSURV-SSC is therefore the diagnostic process (culture only in the absence of lesions, culture and PCR if lesions present).

### 2.4. Component sensitivity calculations

The three SSCs of the French bTB surveillance system were assessed separately, for each species. The component unit sensitivity (CSeU) was first estimated by the sum of the pathways with a positive outcome, itself obtained by multiplying the proportions of the populations in the various risk groups by the probabilities of infection and detection for the branch concerned. Component sensitivity (CSe), corresponding to the probability of detecting at least one bTB-positive wild animal given the presence of bTB at the design prevalence, was then calculated as *CSe* = 1−(1−*CSeU*)^*n*^, considering an arbitrary number of 100 wild animals to be processed per SSC, species and risk area. Finally, the sensitivity of the EC-SSC was estimated specifically for the 2013–2014 hunting season as an example of the application of the model: the CSe was presented for each French “département”, according to the hunting bags of 2013–2014, the risk-levels of the Fig 1A, and take into account the proportion of trained hunters in each area, the proportion of juveniles and adults in the wildlife population, according to species (Table 1) and the probabilities of infection according to species, age class and geographic risk (Table 2).

## Results

The mean, 5^th^ and 95^th^ percentiles of the output distributions of each SSC for unit component sensitivity (CSeU—depending on the probability of infection) are presented in Table 7. The PSURV-SSC had the highest sensitivity, but this component is not applied to all areas and species. The sensitivity of the EC-SSC was higher than that of the SAGIR-SSC, regardless of the species and risk level considered. When considering the probability of infection, which was set according to local risk level, species and age, the effectiveness of the surveillance system increased with increasing probability of infection for the medium and high risk levels (Table 7). Furthermore, the awareness of the hunters, which depends on their training and geographic risk level, affected the sensitivity of the EC-SSC.

**Table 7:** Unit sensitivity (CSeU) for each SSC of the Sylvatub system, by species, age class, and geographic risk (percentage, mean [CI_95%_]).

Species	Low risk level	Medium risk level	High risk level
**EC-SSC**			
**Wild boar**	**UT:** 0.004 [0.001; 0.008]	**UT:** 0.108 [0.031; 0.189]	**UT:** 0.612 [0.026; 1.254]
	**T:** 0.009 [0.004; 0.015]	**T:** 0.187 [0.075; 0.326]	**T:** 0.935 [0.646; 1.232]
**Red deer**	**UT:** 0.003 [0.001; 0.004]	**UT:** 0.055 [0.003; 0.110]	**UT:** 0.187 [0.009; 0.380]
	**T:** 0.007 [0.003; 0.009]	**T:** 0.100 [0.070; 0.131]	**T:** 0.354 [0.262; 0.441]
**Roe deer**	**UT:** 0.002 [0.0008; 0.004]	**UT:** 0.050 [0.002; 0.104]	**UT:** 0.205 [0.127; 0.306]
	**T:** 0.006 [0.003; 0.008]	**T:** 0.074 [0.051; 0.105]	**T:** 0.333 [0.249; 0.416]
**SAGIR-SSC**			
**Wild boar**	0.0004 [0.00008; 0.0009]	0.019 [0.005; 0.042]	0.145 [0.040; 0.320]
**Red deer**	0.0001 [0.00002; 0.0003]	0.011 [0.002; 0.027]	0.045 [0.014; 0.092]
**Roe deer**	0.00009 [0.00003; 0.0002]	**-**	**-**
**Badger**	0.0003 [0.00006; 0.0008]	0.043 [0.012; 0.085]	0.184 [0.054; 0.35]
**PSURV-SSC**			
**Wild boar**	**-**	**-**	1.814 [1.724; 1.907]
**Badger**	**-**	0.75 [0.72; 0.79]	2.154 [2.049; 2.261]

UT: untrained hunter

T: trained hunter

**Fig 5:**
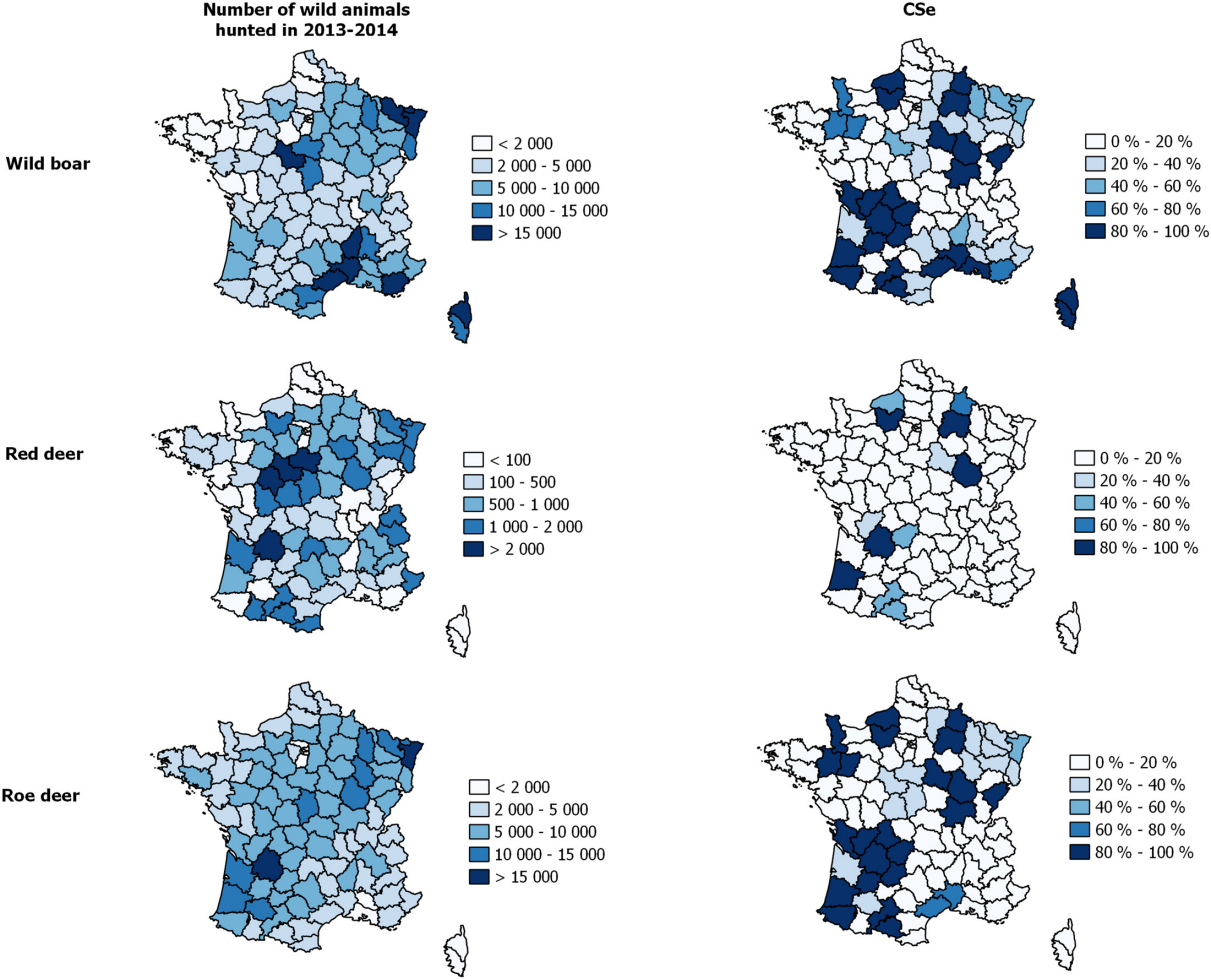
Sensitivity (CSe) of the EC-SSC for wild boar, red deer and roe deer, according to the hunting bags for the 2013–2014 hunting season and the proportion of trained hunters.

We found that the CSe (the probability of detecting at least one bTB-positive animal among 100 wild animals studied over the course of one year) exceeded 50% for the EC-SSC for wild boar (trained hunters) and for the PSURV-SSC for badger and wild boar (Table 8). The number of wild animals actually processed by the EC-SSC exceeded 100 and this surveillance component was therefore more effective than the PSURV-SSC in areas in which hunting was very common (Fig 5, n based on the hunting bags of 2013–2014, by French *département*).

**Table 8:** Component sensitivity (CSe) for each SSC of the Sylvatub system, by species, age class, geographic risk, for 100 animals processed by the SSC, by species and risk level (percentage, mean [CI_95_%]).

Species	Low risk level	Medium risk level	High risk level
**EC-SSC**			
**Wild boar**	**UT:** 0.370 [0.074; 0.744]	**UT:** 10.242 [3.072; 17.244]	**UT:** 45.892 [2.567; 71.689]
	**T:** 0.923 [0.401; 1.458]	**T:** 17.073 [07.210; 27.843]	**T:** 60.901 [47.696; 71.052]
**Red deer**	**UT:** 0.268 [0.076; 0.471]	**UT:** 5.313 [0.260; 10.458]	**UT:** 17.090 [0.886; 31.677]
	**T:** 0.659 [0.328; 0.931]	**T:** 9.513 [6.726; 12.303]	**T:** 29.860 [23.599; 35.749]
**Roe deer**	**UT:** 0.247 [0.076; 0.438]	**UT:** 4.901 [0.240; 9.891]	**UT:** 18.565 [11.934; 26.359]
	**T:** 0.560 [0.321; 0.791]	**T:** 7.153 [4.992; 9.927]	**T:** 28.383 [22.035; 34.056]
**SAGIR-SSC**			
**Wild boar**	0.038 [0.008; 0.096]	2.401 [0.546; 4.081]	13.29 [3.90; 27.42]
**Red deer**	0.0103 [0.002; 0.027]	1.104 [0.233; 2.67]	4.39 [1.41; 8.80]
**Roe deer**	0.009 [0.003; 0.018]	**-**	**-**
**Badger**	0.033 [0.006; 0.078]	4.18 [1.23; 8.18]	16.61 [5.29; 29.36]
**PSURV-SSC**			
**Wild boar**	**-**	**-**	83.94 [82.44; 85.42]
**Badger**	**-**	53.11 [51.36; 54.86]	88.65 [87.38; 89.85]

UT: untrained hunter

T: trained hunter

## Discussion

The surveillance of bTB in wildlife in France involves passive scanning surveillance by carcass examination during hunting and the analysis of animals found dead or dying, together with targeted, risk-based investigations in areas of medium and high risk. The evaluation of this surveillance system is of particular importance, as the persistence of infected wildlife populations in contact with cattle has been shown to hinder the eradication of bTB from domesticated animal populations in some countries [3, 5], and the density and dispersion of susceptible wildlife populations are increasing. The identification of wild maintenance hosts and their effective management is therefore a key determinant of the efficacy of control measures [3, 6, 7], despite the lack of a requirement for mandatory bTB surveillance in wildlife in current EU legislation. The surveillance of bTB is challenging, due to its underlying complex epidemiology and the multiple hosts involved in domestic and wild populations, particularly in wildlife, due to practical constraints imposed.

We used the stochastic scenario tree modelling approach described by Martin *et al*. [13] to estimate the sensitivity of each SSC of the French surveillance system, as it is well adapted to low expected disease prevalences and non-structured probabilistic sampling. This approach can be used to identify and develop efficient surveillance strategies and it enhances communication and collaboration between stakeholders, decision makers and scientists. It provides a transparent and structured approach for decision-making and opportunities for improving the framework, with the acquisition of more accurate data. However, one disadvantage of this method is that it does not take temporal aspects into account (the time interval between infection and TBL development, for example) [31].

With this method, it is possible to include multiple sources of information in the same tree. This is a considerable advantage when not all the data required are available and expert opinion must be sought. In our case, it is difficult to obtain data for bTB prevalence and the densities of susceptible species within a given geographic area, or information about the behaviour of field stakeholders in terms of their participation in the surveillance system and disease awareness. When quantitative information about the key parameters affecting the detection capacity of the SSC was not available, expert advice was sought to reach a consensus, and parameter estimates were described by distributions, relatively wide in some cases, to account for uncertainty. When no consensus between experts could be reached, uniform distributions were used, to take into account the variability or uncertainty in the parameter estimates, which may be considerable for wildlife. The assumptions underlying variable simulations may be biased due to the preconceived opinions of experts, but these expert opinions were nevertheless useful in the absence of epidemiological field survey data. Furthermore, ignoring the effects of parameters that are difficult to quantify (such as disease awareness) would have yielded misleading estimates for the effectiveness of the surveillance system [32].

As information about relative risks of infection and proportion of the population in each risk group were not available for wild populations, it is not possible to use straightforwardly the method described by Martin *et al*. [13]. A two-step approach was so used: first, the relative risks by risk-level area were estimated from cattle data (as there is a good correlation between outbreaks in cattle and in wildlife) and secondly, the Sylvatub data were used to estimate the probabilities of infection according to the species and age class through a standardization procedure. These probabilities may not provide an accurate reflection of the real epidemiological situation in wildlife, but they are nevertheless relevant, as the geographic risk levels for bTB surveillance in wildlife are based, in part, on bTB prevalence in cattle and there is a good correlation between infection rates in wild animals and outbreaks in cattle [9]. The risks of infection for each risk level were then fitted to the Sylvatub data for each species and age. However, only a few infected red and roe deer were detected in France, accounting for the low sensitivity of the surveillance system for these species: the results are thus valid only for the model calculation and assumptions about the risk of infection. The unit sensitivity for an infected animal is available in Supplementary material (S2 File) as it could be useful for adaptation of the results of the model to another epidemiological situation (i.e. another probability of infection according to species, age and geographic area assigned to the mean unit sensitivity).

PSURV-SSC and EC-SSC had the highest CSe for detecting at least one infected wild animal at the design prevalence, regardless of species. The SAGIR-SSC had a very low CSe, whatever the species considered, particularly at low (CSe < 1%) and medium (CSe < 5%) levels of risk. Furthermore, fewer animals than planned (100 for this model) may be processed in reality for this surveillance component. The reinforcement of the SAGIR network increases the sensitivity of this SSC, particularly at high levels of risk (CSe between 5% and 20%). This component remains relevant, as it is the only surveillance component applied in summer (no hunting is generally allowed in the summer for wild boar and deer, so the probability of detection by hunting is zero during this period): the seasonality of surveillance should therefore also be taken into account when assessing the overall effectiveness of the surveillance system.

Post-mortem examination (ability to detect TBLs) has a low sensitivity, but training to increase the awareness of hunters and being in a high risk area increased the sensitivity of the EC-SSC. The experts considered the efficacy of post-mortem inspection to be greater for deer than for wild boar, because the TBLs are often located on cervical lymph nodes in wild boars and are, therefore, difficult for hunters to detect during their examination of the carcass (Table 4). These assumptions are consistent with the findings of several studies showing the sensitivity of post-mortem inspection to be low [33, 34, 35]. Nevertheless, we found that the EC-SSC had a high CSe, due to the large numbers of wild animals killed during hunting and subjected to post-mortem examination, even if this post-mortem examination and the submission of suspect carcasses are voluntary (Fig 5). The sensitivity of active surveillance was also high, because the collection of animals was not influenced by the probability of the animals having TBLs or the ability of hunters to detect these lesions (systematic collection and analyses) and because the samples are collected around bTB cattle outbreaks.

The diagnostic process currently consists of systematic culture, with PCR if TBLs are detected. However, tuberculous lesions may be non-specific in appearance, particularly at early stages of infection, limiting the sensitivity of the overall diagnostic process (Table 6). The sensitivity of culture and PCR on wild populations have been estimated by expert opinions compared to sensitivity on cattle populations [21], which were however estimated from suspect samples and are not representative of the sensitivity of the tests in the whole population. The tests used have been considered to be independent, whereas there is actually a biological dependence that could lead to a smaller gain in sensitivity [34]. However, estimation of the sensitivity covariance would require comparison with a third reference test, but no such data were available for this study. Furthermore, culture is the reference test for bovine tuberculosis, but its sensitivity is relatively low for wild populations, due to conditions in the field and the potential contamination of samples. This method is also time-consuming and costly. A serological test for the detection of *M*. *bovis* antibodies was recently evaluated in wild boars and may provide a suitable alternative for bTB screening in wild boar populations [36, 9].

In this study, we assessed the effectiveness of the current bTB surveillance system for wildlife in France. Only a few studies have evaluated the sensitivity of bTB surveillance in wildlife, and most have focused on farmed wild species, such as deer [34, 35], for which social behaviour may influence the probability of infection and for which tuberculin tests could be used to screen living animals and meat inspections could be performed at slaughterhouses, increasing the overall sensitivity of surveillance. These surveillance systems are less sensitive to behavioural effects (such as the participation of stakeholders in cases of passive surveillance) and field conditions (degradation and contamination of carcasses or samples). The ability to detect TBLs on post-mortem inspection, estimated on the basis of expert opinion and data from cattle, was therefore considered to be higher in these previous studies than in our model, as meat inspection is conducted in better conditions and is more detailed than carcass examination by a hunter.

Further investigations are required for assessment of the overall sensitivity of the entire surveillance system, but data for overall badger density are not available (localised studies have been conducted, but the results obtained cannot be extrapolated to other geographic areas). Furthermore, specificity is also a relevant issue, as the collection of non-tuberculous wild animals could lead to unnecessary laboratory costs. The government and hunters’ association should cover the costs of surveillance activities, with compensation provided to field stakeholders for their participation. Assessments of the cost-effectiveness of the surveillance system would thus be useful, to confirm the advantages of implementing one or more components in different areas, for the various stakeholders (this has already been done in some countries [15]).

This was the first quantitative evaluation of the sensitivity of the bTB surveillance system in wildlife in France. The effectiveness of the Sylvatub system is difficult to estimate, because it is dependent on many factors, such as the probability of infected animals having lesions, the disease awareness of hunters and their willingness to report suspected cases of infection. The inclusion of behavioural effects (the hunters’ awareness of the disease, and their willingness to report their suspicions if TBLs are detected) might introduce a bias, as these effects are difficult to estimate, but the results are relevant for hunters and veterinary authorities wishing to know the actual efficacy of bTB surveillance in free-ranging wildlife as a function of geographic area and species. They could also provide support for decision-making processes concerning the enhancement of surveillance strategies. Further investigations should be performed to identify key factors influencing the CSe, to determine how the efficacy of the surveillance system could be improved, given that some biological factors cannot be influenced by surveillance strategies (such as the probability of TBLs being visible on infected wild animals) [15, 37], and to assess the cost-effectiveness of each SSC. The results obtained for this model could encourage stakeholders to participate in the surveillance system, improving its efficiency, but it may be difficult to standardise the participation of hunters and to integrate their role into a formal surveillance strategy. Indeed, information flow, awareness, local acceptance and incentives should be taken into account when trying to improve case reporting and, thus, the effectiveness and sustainability of the surveillance system.

## Supporting Information

S1 File Description of the Delphi approach and of the questionnaire sent to experts. (DOCX)

S2 File Unit sensitivity for an infected animal (SeU) for each SSC of the Sylvatub system, by species, age class and geographic risk. (DOCX)

S3 File ARRIVE Checklist. (PDF)
