# Supplementary material for: Sensitivity of Bovine Tuberculosis Surveillance in Wildlife in France: A Scenario Tree Approach
Source: PLoS One. 2015 Oct 30;10(10):e0141884. doi: 10.1371/journal.pone.0141884 (PMC4627846; doi:10.1371/journal.pone.0141884)
Supplement: S1 File — (DOCX) [file pone.0141884.s001.docx]

**Sensitivity of bovine tuberculosis surveillance in wildlife in France: a scenario tree approach**

**J. RIVIERE, Y. LE STRAT, B. DUFOUR, P. HENDRIKX**

**s1 file:**

**DELPHI APPROACH**

**Description of the experts**

|  | Trainer in carcass examination | Stakeholder of the SAGIR network | Trainer in carcass examination and stakeholder of the SAGIR network | Veterinary surgeon | Total |
| --- | --- | --- | --- | --- | --- |
| National | - | - | - | - | 5 |
| *Départemental* |  |  |  |  |  |
| Low risk | 1 | 3 | 2 | - | 6 |
| Medium risk | 1 | 0 | 1 | - | 2 |
| High risk | - | 1 | 1 | 1 | 3 |

**Questionnaire**

**QUESTION 1: General information**

Please provide the following information before completing the questionnaire. Your answers will remain anonymous.

*Surname: text zone

*First name: text zone

*Institution (e.g. FDC, ONCFS): text zone

*Post held (e.g. trainer in the initial examination of game animals, ITD SAGIR): text zone

**Département* in which active: Pull-down menu

*Telephone number: 10-digit number

*E-mail address: text zone

*Other information: text zone

**QUESTION 2: Age classes; classification of subadults**

Classically, three age groups are used for wildlife: adult, subadult and juvenile. We have decided to simplify the analysis by using only two age groups: adult and juvenile. The age of a wild animal will be used to model several probabilities, such as the probability of an animal being killed by hunters and the probability of that animal bearing lesions suggestive of tuberculosis.

**Please indicate with which age group you would combine the subadult category, for each of the following species:**

| **Wild boar** | **Deer (red deer/roe deer)** | **Badger** |
| --- | --- | --- |
| Adult  Juvenile  Don’t know | Adult  Juvenile  Don’t know | Adult  Juvenile  Don’t know |

Comments: text zone

**QUESTION 3: Presence of macroscopic lesions suggestive of tuberculosis**

The probability of a wild animal presenting macroscopic lesions* suggestive of tuberculosis (of the abscess type) depends on the species and age of the animal and its infection status (infected with the causal agent of tuberculosis or uninfected with this agent but presenting a condition involving macroscopic lesions similar to those of tuberculosis).

**Macroscopic lesions are abnormalities that can be detected on visual examination by the hunter.*

**What do you think is the probability of an animal infected or not infected with the causal agent of tuberculosis presenting macroscopic lesions suggestive of tuberculosis, as a function of the species and age of the animal?**

| **Species** | **Age** | **Animal infected with the causal agent of tuberculosis** | **Animal not infected with the causal agent of tuberculosis but potentially presenting macroscopic lesions due to another pathogenic agent** |
| --- | --- | --- | --- |
| **Wild boar** | Juvenile |  |  |
|  | Adult |  |  |
| **Red deer** | Juvenile |  |  |
|  | Adult |  |  |
| **Roe deer** | Juvenile |  |  |
|  | Adult |  |  |
| **Badger** | Juvenile |  |  |
|  | Adult |  |  |

*Please indicate the value that you think is most likely.*

*If you wish, you can indicate an interval, as follows: “lower limit; most likely value; upper limit”. For example, for a probability between 5% and 30%, with a most likely value of 10%, you should write 5; 10; 30.*

Comments: text zone

**QUESTION 4: Detection of macroscopic lesions suggestive of tuberculosis on animals killed by hunters**

The probability of detecting lesions suggestive of tuberculosis on an animal killed by hunters depends on two principal factors:

- The species of animal and the type of lesion (small and localised or extensive and potentially affecting several organs)

- The expertise of the hunter carrying out the carcass examination (trained or untrained in the performance of this examination, frequency of observation of lesions suggestive of tuberculosis, varying with the risk level of the geographic zone). This level of expertise influences principally the capacity to detect lesions suggestive of tuberculosis, the declaration of suspected cases when suggestive lesions are detected and the quality of sampling for tests.

*NB: The risk levels of the various geographic regions (1, 2, 3) are those defined by Sylvatub.*

**What would you say is the probability of a hunter not trained in carcass examination detecting lesions suggestive of tuberculosis, for each of the species indicated, in zones with the risk level indicated?**

|  | **Risk level 1** | **Risk level 2** | **Risk level 3** |
| --- | --- | --- | --- |
| **Wild boar** |  |  |  |
| **Red deer** |  |  |  |
| **Roe deer** |  |  |  |

*Please indicate the value that you think is most likely.*

*If you wish, you can indicate an interval, as follows: “lower limit; most likely value; upper limit”. For example, for a probability between 5% and 30%, with a most likely value of 10%, you should write 5; 10; 30.*

Comments: text zone

**What would you say is the probability of a hunter trained in carcass examination detecting lesions suggestive of tuberculosis, for each of the species indicated, in zones with the risk level indicated?**

|  | **Risk level 1** | **Risk level 2** | **Risk level 3** |
| --- | --- | --- | --- |
| **Wild boar** |  |  |  |
| **Red deer** |  |  |  |
| **Roe deer** |  |  |  |

*Please indicate the value that you think is most likely.*

*If you wish, you can indicate an interval, as follows: “lower limit; most likely value; upper limit”. For example, for a probability between 5% and 30%, with a most likely value of 10%, you should write 5; 10; 30.*

Comments: text zone

**QUESTION 5: Detection of dead and dying animals by members of the SAGIR network**

We think that the probability of a dead or dying animal being detected by a member of the SAGIR network depends on the behaviour of the species concerned.

**What do you think is the probability of a dead or dying wild animal being detected by a member of the SAGIR network, for the species listed?**

| **Wild boar** |  |
| --- | --- |
| **Red deer** |  |
| **Roe deer** |  |
| **Badger** |  |

*Please indicate the value that you think is most likely.*

*If you wish, you can indicate an interval, as follows: “lower limit; most likely value; upper limit”. For example, for a probability between 5% and 30%, with a most likely value of 10%, you should write 5; 10; 30.*

Comments: text zone

**QUESTION 6: Collection of a dead or dying animal by a member of the SAGIR network**

We think that the probability of a dead or dying animal being collected by a member of the SAGIR network depends on several factors:

- The size of the animal, and thus its species and age,
- The state of decomposition of the animal,
- The degree of awareness of the agent in the field, which may vary with the level of tuberculosis risk in the *département* concerned,
- The laboratory analyses being paid for by the agents in the field, as part of the normal functioning of the SAGIR network (no indemnities paid), and regardless of the risk level for roe deer.

In areas with a risk level of 2 or 3, more support for the collection of dead or dying animals has been requested by Sylvatub, with the payment of indemnities for the collection of a certain number of animals. For areas with a risk level of 1, the SAGIR network will continue to function as normal (no indemnities paid).

*NB: The risk levels (1, 2, 3) are those defined by Sylvatub.*

**What do you think is the probability of a dead or dying animal being collected by a member of the SAGIR network, as a function of its size (species and age) and the geographic area (defined in terms of Sylvatub risk levels)?**

| **Species** | **Age** | **Risk level 1** | **Risk level 2** | **Risk level 3** |
| --- | --- | --- | --- | --- |
| **Wild boar** | Juvenile |  |  |  |
|  | Adult |  |  |  |
| **Red deer** | Juvenile |  |  |  |
|  | Adult |  |  |  |
| **Roe deer** | Juvenile |  |  |  |
|  | Adult |  |  |  |
| **Badger** | Juvenile |  |  |  |
|  | Adult |  |  |  |

*Please indicate the value that you think is most likely.*

*If you wish, you can indicate an interval, as follows: “lower limit; most likely value; upper limit”. For example, for a probability between 5% and 30%, with a most likely value of 10%, you should write 5; 10; 30.*

Comments: text zone

*-----*

Thank you very much for agreeing to complete this questionnaire. We may contact you again in the next few days, by e-mail or by telephone, if any clarification of your responses is required. We remain at your disposal for any questions you may have or for any additional information about this study.
