# Supplementary material for: Sensitivity of Bovine Tuberculosis Surveillance in Wildlife in France: A Scenario Tree Approach
Source: PLoS One. 2015 Oct 30;10(10):e0141884. doi: 10.1371/journal.pone.0141884 (PMC4627846; doi:10.1371/journal.pone.0141884)
Supplement: S2 File — (DOCX) [file pone.0141884.s002.docx]

**Sensitivity of bovine tuberculosis surveillance in wildlife in France: a scenario tree approach**

**J. RIVIERE, Y. LE STRAT, B. DUFOUR, P. HENDRIKX**

**S2 File:**

**Unit sensitivity for an infected animal (SeU)**

**Unit sensitivity (SeU) for each SSC of the Sylvatub system, by species, age class, and geographic risk (percentage, mean [CI _95%_])**

| **Species** | **Age class** | **Low risk level (%)** | **Medium risk level (%)** | **High risk level (%)** |
| --- | --- | --- | --- | --- |
| **EC-SSC** | | | | |
| **Wild boar** | **Juvenile** | **UT:** 5.0 [1.0; 10.5]  **T:** 12.7 [5.2; 21.7] | **UT:** 10.8 [2.9; 20.1]  **T:** 18.7 [7.1; 34.3] | **UT:** 21.7 [1.2; 47.3]  **T:** 32.9 [21.2; 47.8] |
|  | **Adult** | **UT:** 5.2 [1.1; 10.7]  **T:** 13.1 [5.6; 21.0] | **UT:** 11.1 [3.1; 20.0]  **T:** 19.3 [7.6; 33.9] | **UT:** 22.3 [1.2; 45.8]  **T:** 33.9 [23.5; 45.9] |
| **Red deer** | **Juvenile** | **UT:** 17.1 [5.2; 30.0]  **T:** 42.2 [21.2; 59.0] | **UT:** 25.6 [1.3; 51.8]  **T:** 46.7 [32.5; 61.2] | **UT:** 30.7 [1.5; 62.1]  **T:** 58.0 [42.4; 72.1] |
|  | **Adult** | **UT:** 16.5 [5.0; 28.8]  **T:** 40.6 [20.1; 57.8] | **UT:** 24.6 [1.3; 50.2]  **T:** 44.9 [31.0; 59.1] | **UT:** 29.6 [1.5; 60.3]  **T:** 55.8 [40.5; 70.1] |
| **Roe deer** | **Juvenile** | **UT:** 15.1 [4.3; 26.7]  **T:** 34.2 [19.9; 48.0] | **UT:** 22.9 [1.0; 46.5]  **T:** 33.2 [23.2; 47.1] | **UT:** 32.1 [19.9; 48.0]  **T:** 52.3 [39.0; 64.5] |
|  | **Adult** | **UT:** 15.2 [4.3; 27.2]  **T:** 34.5 [19.9; 49.1] | **UT:** 23.1 [1.1; 47.8]  **T:** 33.5 [22.8; 47.8] | **UT:** 32.4 [19.7; 48.4]  **T:** 52.9 [38.7; 66.8] |
| **SAGIR-SSC** | | | | |
| **Wild boar** | **Juvenile** | 0.67 [0.06; 1.91] | 2.48 [0.51; 6.01] | 5.74 [1.06; 13.78] |
|  | **Adult** | 0.45 [0.04; 1.31] | 6.01 [0.36; 4.13] | 5.30 [1.00; 13.05] |
| **Red deer** | **Juvenile** | 0.70 [0.06; 2.13] | 4.77 [0.84; 12.02] | 8.11 [2.59; 16.90] |
|  | **Adult** | 0.74 [0.07; 2.28] | 6.31 [0.61; 17.36] | 8.11 [2.29; 18.04] |
| **Roe deer** | **Juvenile** | 0.60 [0.14; 1.40] | **-** | **-** |
|  | **Adult** | 0.61 [0.13; 1.41] | **-** | **-** |
| **Badger** | **Juvenile** | 0.30 [0.02; 0.95] | 1.39 [0.09; 4.38] | 5.57 [1.57; 10.81] |
|  | **Adult** | 0.45 [0.05; 1.21] | 5.55 [1.60; 10.53] | 5.78 [1.61; 11.37] |
| **PSURV-SSC** | | | | |
| **Wild boar** | **Juvenile** | **-** | **-** | 64.9 [61.3; 68.4] |
|  | **Adult** | **-** | **-** | 65.9 [62.7; 69.1] |
| **Badger** | **Juvenile** | **-** | 58.9 [55.4; 62.5] | 58.9 [55.4; 62.5] |
|  | **Adult** | **-** | 59.9 [56.9; 62.9] | 59.9 [56.9; 62.9] |
